# Predicting small molecule binding pockets on diacylglycerol kinases using chemoproteomics and AlphaFold†

**DOI:** 10.1039/d3cb00057e

**Published:** 2023-05-15

**Authors:** Roberto Mendez, Minhaj Shaikh, Michael C. Lemke, Kun Yuan, Adam H. Libby, Dina L. Bai, Mark M. Ross, Thurl E. Harris, Ku-Lung Hsu

**Affiliations:** a Department of Chemistry, University of Virginia Charlottesville Virginia 22904 USA ken.hsu@austin.utexas.edu +1 434-297-4864; b Department of Pharmacology, University of Virginia School of Medicine Charlottesville Virginia 22908 USA; c Department of Molecular Physiology and Biological Physics, University of Virginia Charlottesville Virginia 22908 USA; d University of Virginia Cancer Center, University of Virginia Charlottesville VA 22903 USA

## Abstract

Diacylglycerol kinases (DGKs) are metabolic kinases involved in regulating cellular levels of diacylglycerol and phosphatidic lipid messengers. The development of selective inhibitors for individual DGKs would benefit from discovery of protein pockets available for inhibitor binding in cellular environments. Here we utilized a sulfonyl-triazole probe (TH211) bearing a DGK fragment ligand for covalent binding to tyrosine and lysine sites on DGKs in cells that map to predicted small molecule binding pockets in AlphaFold structures. We apply this chemoproteomics-AlphaFold approach to evaluate probe binding of DGK chimera proteins engineered to exchange regulatory C1 domains between DGK subtypes (DGKα and DGKζ). Specifically, we discovered loss of TH211 binding to a predicted pocket in the catalytic domain when C1 domains on DGKα were exchanged that correlated with impaired biochemical activity as measured by a DAG phosphorylation assay. Collectively, we provide a family-wide assessment of accessible sites for covalent targeting that combined with AlphaFold revealed predicted small molecule binding pockets for guiding future inhibitor development of the DGK superfamily.

## Abstract 2

We provide a family-wide assessment of accessible sites for covalent targeting that combined with AlphaFold revealed predicted small molecule binding pockets for guiding future inhibitor development of the DGK superfamily.

## Graphical abstract

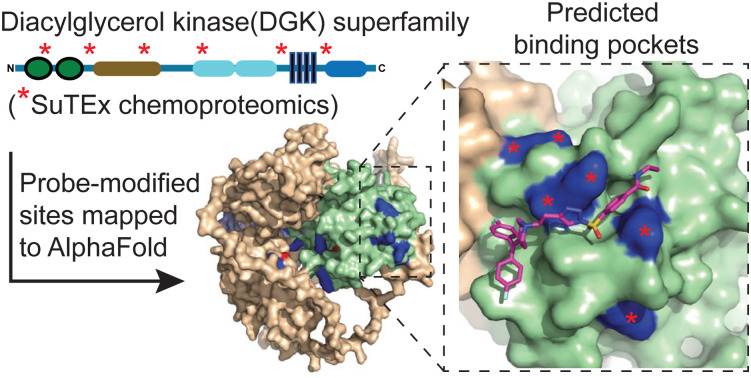

## Introduction

**Fig. 1:**
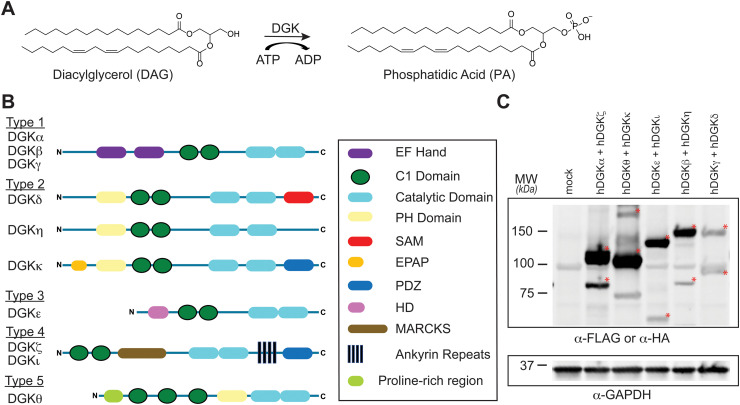
Activity, regulation, and recombinant expression of the mammalian diacylglycerol kinase (DGK) superfamily. (A) DGKs catalyze ATP-dependent phosphorylation of diacylglycerols to biosynthesize phosphatidic acid. DGK-regulated lipids modulate cognate protein receptors through changes in localization, activation, and protein–protein interactions. (B) DGKs are multidomain lipid kinases that differ principally in composition of regulatory domains outside of the conserved catalytic domain. (C) Co-expression of recombinant human DGK proteins in HEK293T cells for chemical proteomic evaluation. Expression of recombinant DGKs was detected by western blots using anti-FLAG antibodies except for DGKζ and DGKι, which were detected with anti-HA antibody. Equivalent protein loading was confirmed by anti-GAPDH. All data shown are representative of *n* = 3 biologically independent experiments. Recombinant proteins are highlighted by red asterisk.

Diacylglycerol kinases (DGKs) are multidomain lipid kinases that catalyze ATP-dependent phosphorylation of diacylglycerol (DAG) to generate phosphatidic acid (PA).^1–4^ DAGs play important roles in lipid metabolism and signaling by serving as a secondary messenger, membrane constituent, and metabolic building block.^5–8^ PA mediates signaling through binding to cognate receptor proteins and can serve as a precursor for lysophospholipids and glycero(phospho)lipids.^9–11^ DGK activity is implicated in regulating the cellular levels and fatty acyl composition of DAG and PA although the molecular basis of this specificity remains an active area of investigation (Fig. 1(A)). Recent efforts using chemical proteomics and lipidomics support crosstalk between catalytic and regulatory domains of DGKs as a potential mechanism for mediating lipid substrate selectivity.^12^

Mammals express 10 known DGK isoforms that share a conserved lipid kinase domain (split into the DAGKc and DAGKa regions) and a minimum of two C1 domains (tandem C1A and C1B). DGKs are differentiated principally by regulatory domains that are involved in regulation of DGK activation (EF hand motifs), membrane localization (PH domain), and protein–protein interactions^1^ (PDZ domain; Fig. 1(B)). DGK metabolism and signaling can be regulated through cell type- and tissue-specific expression.^1–4^ For example, DGKα and DGKζ expression is enriched in T cells compared with other cell and tissue types.^13^ Consequently, deficiency of these DGK isoforms in mice result in enhanced Ras and mitogen-activated protein kinase (MAPK) activation in response to T cell receptor (TCR) stimulation.^14,15^ These findings support DGKs as metabolic ‘checkpoints’ for TCR-MAPK signaling by restricting available DAG messengers for signal transduction. Overactive DGKα and/or DGKζ has been implicated in defective tumor immune responses, and development of selective inhibitors against these T cell-specific DGKs is being pursued as an immunotherapy strategy in cancer.^13–27^

Recently, we utilized ATP acyl phosphate activity-based probes to identify DGK sites important for mediating substrate and inhibitor binding in native lysates.^28^ ATP acyl phosphates are used for global activity-based profiling of ATP-binding pockets on kinases and ATPases. This activity-based probe is directed to kinase active sites by ATP recognition followed by covalent binding of lysines proximal to the acyl phosphate electrophile.^29,30^ Competitive studies with free ATP identified ATP substrate-binding sites in the catalytic domain of representative members of all five DGK subtypes.^28^ Furthermore, this study implicated the cysteine rich (C1) domain of rat DGKα (K237), human DGKζ (K323), and human DGKθ (K202) in recognition of the ATP acyl phosphate probe. DGKs, with the exception of DGKβ and DGKγ, contain atypical C1 domains of poorly defined function that are distinct from typical counterparts used by protein kinase C (PKC) for DAG-mediated translocation and activation.^31,32^ Functional lipidomics identified C1 domains as important mediators of DAG substrate specificity of DGKs; this C1-mediated specificity could be exchanged between type 1 DGK family members by protein engineering.^12,28^

The chemoproteomic studies to date have been performed largely in cell lysates.^28^ While informative, the binding profiles obtained *in vitro* may not reflect protein states that are subject to complex regulation in cellular environments. We developed a first-generation covalent probe, TH211, capable of functional profiling of DGKα in live T cells.^33^ The TH211 probe contains a sulfonyl-triazole electrophile (SuTEx^34^) that reacts with tyrosine and lysine sites when directed to DGK active sites with the kinase binding element RF001 derived from the DGK inhibitor ritanserin.^35–38^ The prominent TH211 binding to the C1 domain of DGKα in T cells coupled with impaired biochemical activity when TH211-modified C1 sites are mutated support potential allosteric regulation through this domain. Whether probe binding to C1 domains of other DGK members occurs in cellular environments, which would support a more general as opposed to DGKα-specific regulatory mechanism, is currently unknown.

Here we established cellular binding profiles for all members of the DGK superfamily using TH211.^33^ Treatment of recombinant DGK expressing cells with TH211 facilitated identification of tyrosine and lysines sites that could be mapped onto available AlphaFold structures to predict small molecule binding regions across the DGK superfamily. We applied this chemoproteomic-AlphaFold approach to investigate cellular binding of TH211 to DGK chimera proteins with exchanged C1 domains between subtypes to further expand our understanding of crosstalk between regulatory and catalytic domains in molecular recognition of DGKs.

## Results

### Chemoproteomic profiling of the DGK superfamily *in situ*

**Fig. 2:**
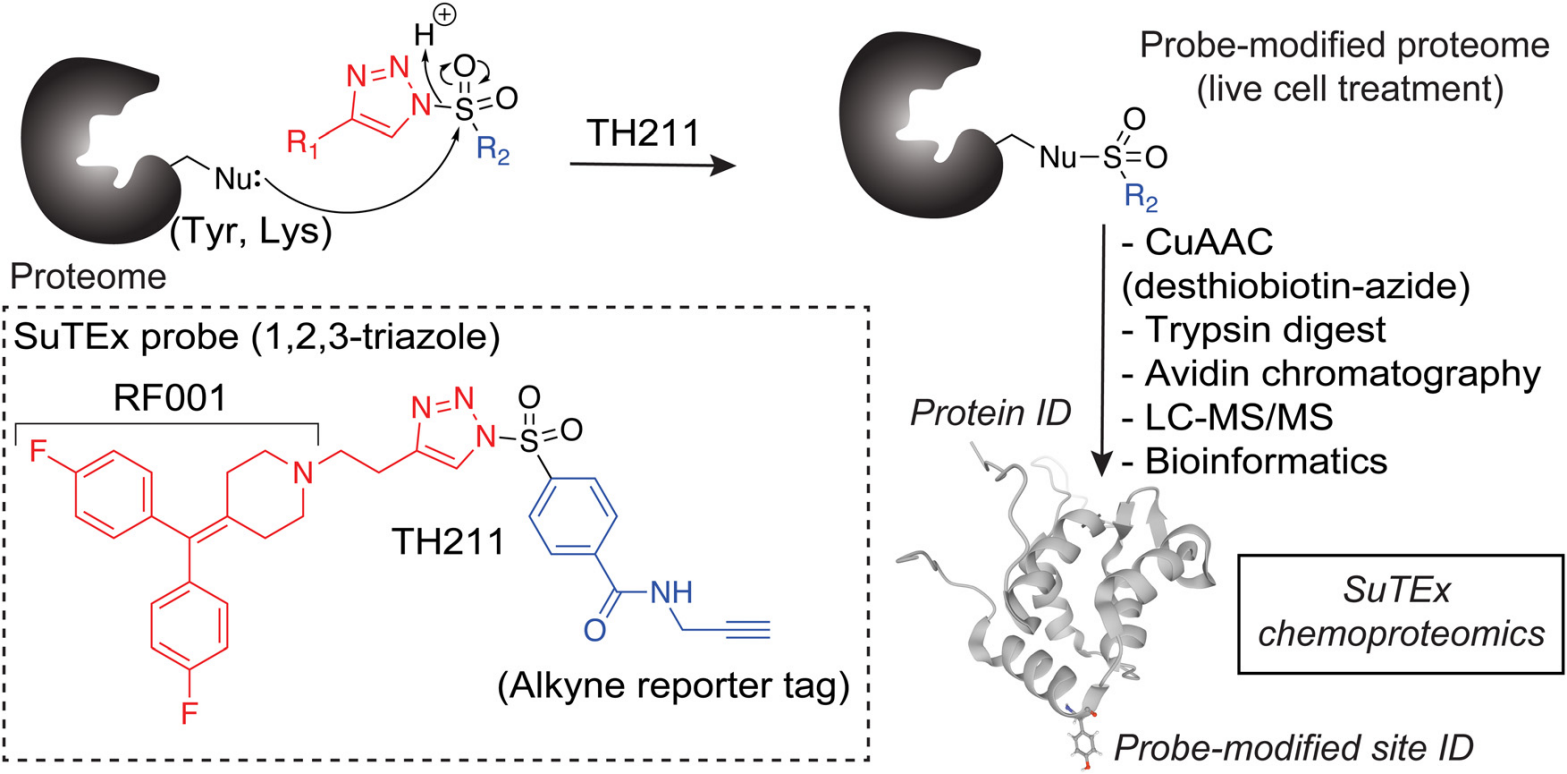
Chemoproteomic profiling of the DGK superfamily in live cells. 1,2,3-Sulfonyl-triazoles modify Tyr and Lys sites, with a preference for the former residue, on proteins through sulfur-triazole exchange chemistry (SuTEx) chemistry. Proteins modified by the SuTEx probe TH211 are tagged with an alkyne group to facilitate copper-catalyzed azide–alkyne cycloaddition (CuAAC) conjugation of a desthiobiotin-azide enrichment handle. Desthiobiotin-tagged proteins are digested with trypsin protease to produce probe-modified peptides that are enriched by avidin chromatography followed by LC–MS/MS analysis. Bioinformatics can identify probe-modified peptide sequences and site of probe modification that can be used to infer covalent binding of SuTEx probes to intact proteins from lysate and live cell studies.

To assess covalent probe binding across the entire DGK superfamily, we chose a recombinant overexpression system in HEK293T cells because detection of endogenous members would require multiple cell lines to achieve family-wide coverage. TH211 was chosen for chemical proteomic studies because of prior demonstration of TH211 binding to functional sites in the C1 and catalytic domain of DGKα in cells^33^ (Fig. 2). We confirmed TH211 blocks catalytic activity of recombinant DGKα and DGKζ, which supports functional binding activity of TH211 across multiple DGK subtypes (Fig. S1, ESI†). The ability to perform TH211 labeling *in situ* was an important criterion for probe selection to enable access to DGK activity states under dynamic regulation in cells.

SILAC light (L) and heavy (H) HEK293T cells were transiently transfected with plasmids encoding recombinant DGKs of the entire mammalian superfamily. We performed chemical proteomic studies on cells co-expressing DGK pairs to (i) understand covalent probe binding to multiple DGKs in a cellular environment, and (ii) streamline our workflow to minimize sample-to-sample variations. DGK isoform pairs were chosen based on distinct subtype classification and gel-resolvable molecular weights to permit facile verification of expression by western blots. Based on these criteria, the selected pairs for chemoproteomic evaluation included co-expression of DGKα (type 1):DGKζ (type 4), DGKκ (type 2):DGKθ (type 5), DGKι (type 4):DGKε (type 3), DGKβ (type 1):DGKη (type 2), and DGKγ (type 1):DGKδ (type 2).

First, we overexpressed DGK pairs in HEK293T cells and verified comparable protein expression of recombinant DGKs in light and heavy cells by western blot (α-FLAG or α-HA antibodies, Fig. 1(C) and Fig. S2, ESI†). Next, DGK-expressing SILAC HEK293T cells were treated with either DMSO vehicle or TH211 (50 μM, 2 h, 37 °C). Following probe labeling, cells were lysed, proteomes conjugated to desthiobiotin-azide by copper-catalyzed azide–alkyne cycloaddition (CuAAC). Proteomes were subjected to trypsin protease cleavage followed by avidin chromatography enrichment of probe-modified peptides. Liquid chromatography-tandem mass spectrometry (LC–MS/MS) analysis facilitated identification of TH211 modified sites on DGKs covalently bound in live cells (Fig. 2). Our mixing conditions accounted for specific enrichment of probe-modified sites on DGKs (L-TH211/H-DMSO) as well as a 1 : 1 mixing control condition (L-TH211/H-TH211) as previously described.^33^

Probe-modified sites specifically enriched and detected by data-dependent acquisition were identified by a SILAC ratio (SR) >5 for TH211 probe (L)- compared with DMSO (H)-treated samples. These peptides were further evaluated for quality control confidence criteria that include a Byonic search algorithm score of ≥500, 1% protein false discovery rate (FDR), and mass accuracy (≤5 ppm) as previously described.^33,39,40^ We found significant correlations between individual biological replicates across the chemoproteomic samples evaluated; data for probe-modified peptides from individual DGK proteins detected and replicate correlation coefficients can be found in Table S1A and B (ESI†).

### Location of covalent binding sites detected on human DGKs

Our chemical proteomic studies revealed TH211 binding to tyrosine (Tyr) and lysine (Lys) residues across all 10 DGK isoforms. We did not detect probe-modified peptides on endogenous DGKs in TH211-treated, mock transfected HEK293T except for Y148 and Y669 on DGKα, supporting assignment of probe-modified sites principally to recombinant proteins (Table S1C, ESI†). The covalent labeling profiles across all quantified sites showed a more comparable detection of modified Tyr and Lys residues (Y/K ratio ∼1.3), which agreed with our previous finding that 1,2,3-sulfonyl-triazoles, including TH211, showed increased Lys binding activity compared with 1,2,4-SuTEx counterparts.^33,34^ The lower natural abundance of Tyr compared with that of Lys^41^ was reflected in the frequency of modified residues that was dependent, to some degree, on amino acid composition of DGKs (average Tyr and Lys composition of ∼2 and 6%, respectively; Table S1D, ESI†).

As expected, prominent probe binding was observed in the catalytic domain of all DGKs evaluated (Table S1B, ESI†). We detected at least a single modified Tyr or Lys in the catalytic domain and in some instances, multiple binding events to the DAGKc and DAGKa regions of DGKs (*e.g.*, DGKζ, DGKδ, DGKι; Table S1B, ESI†). We also identified trends in TH211 binding at regulatory domains that support potential differences in molecular recognition between DGK members. For example, probe-modified sites, and Tyr specifically, were identified only on EF hands of DGKα among the type 1 DGKs (Y169, Table S1B, ESI†). The pattern of Tyr modifications in C1 domains across the DGKs also matched our previous finding that probe binding is prominent in C1A and C1A/C1B of DGKα and DGKγ, respectively.^12^ In contrast, probe-modified Lys were frequent in C1 domains of DGKs members outside of type 1 except for DGKκ, DGKε and DGKη that were devoid of probe binding in these regions (Table S1B, ESI†).

Importantly, we detected a comparable number of TH211-modified sites on DGKβ and DGKη despite a drastic difference in recombinant protein expression levels in co-expressed HEK293T cells (Fig. 1(C) and Table S1B, ESI†). To test whether co-expression affected the resulting site detection on recombinant DGKs, we performed chemoproteomic evaluation of HEK293T cells expressing recombinant DGKβ alone and found negligible differences in the number of sites identified compared with DGKβ/DGKη co-expressed cell counterparts (Table S1B and E, ESI†). These data support TH211 reactivity at DGK sites that is not dependent solely on protein expression differences from co-expressed pairs.

In contrast to previous ATP acyl phosphate studies,^37^ we detected covalent binding in regulatory domains orthogonal to C1 and catalytic domains that could serve as target sites for covalent DGK inhibitor development. For example, several probe-modified sites were detected in the ankyrin repeats of type 4 DGKs and the Ras-association domain of DGKθ. Additional probe-modified sites of interest included covalent binding to the PH domain of DGKκ (K286) and the SAM domain of DGKδ (K1191, Table S1B, ESI†). The presence of probe-modified Tyr and Lys in the peptide region between the DAGKc and DAGKa of DGK-δ, -κ and -η should enable future investigations of this poorly annotated region that differentiate type 2 DGKs from the rest of the superfamily (Table S1B, ESI†).

### Identifying predicted TH211 binding pockets on DGK AlphaFold structures

**Fig. 3:**
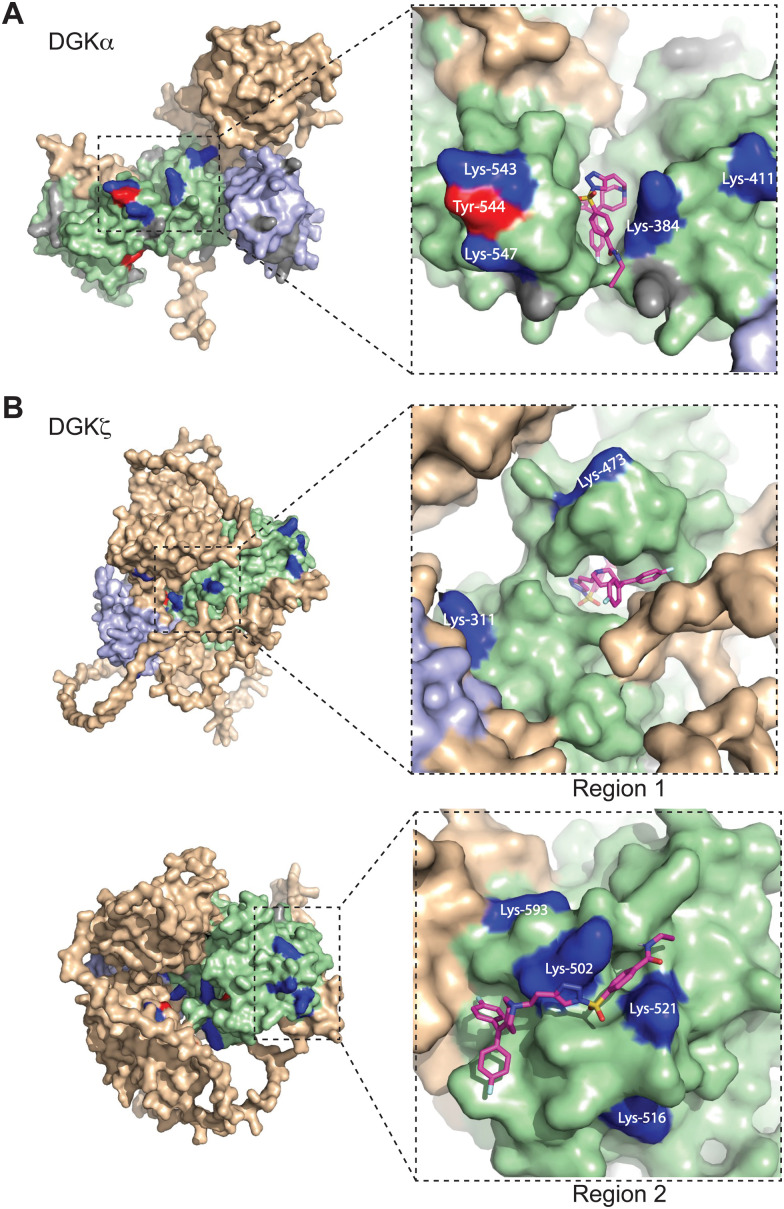
Covalent binding to predicted pockets of DGKα and DGKζ. Binding sites detected from TH211 treatments of recombinant DGK overexpressed-HEK293T cells and quantitative chemical proteomics are mapped onto AlphaFold structures (DGKα: AF-P23743-F1; DGKζ: AF-Q13574-F1). Cells were treated with TH211 (50 μM) for 2 h at 37 °C. The covalent binding profiles of DGKα and DGKζ are shown here. The remaining DGK protein AlphaFold structures can be found in Fig. S3 (ESI†). (A) DGKα with inset showing expanded region of TH211 docked on Lys-384 in the predicted binding pocket. (B) DGKζ with inset showing expanded region with TH211 docked on Lys-473. A second predicted binding pocket of DGKζ with inset showing expanded region of TH211 docked on Lys-502. C1A and C1B domains are shaded light blue, and the catalytic domain (DAGKc and DAGKa regions) is shaded in light green. Probe modified Lys and Tyr are shown in dark blue and red, respectively. Lys and Tyr residues confidently predicted by AlphaFold (“Confident” and “Very Confident” predictions, pLDDT > 70) but not modified by our probe are shown in gray. Lys and Tyr residues predicted less confidently (“Low” and “Very Low” predictions, pLDDT < 70) are not highlighted and were not included in the analysis. All data shown are representative of *n* = 3 biologically independent experiments. Predicted structures were visualized using PyMOL (Version 2.6; https://pymol.org).

Next, we sought to translate the individual TH211-modified sites identified by chemical proteomics into binding regions across the DGK superfamily. Given the lack of available full-length structures for mammalian DGKs, we used AlphaFold^42,43^ to map TH211-modified sites onto predicted binding pockets in these lipid kinases. Tyr and Lys residues that were confidently predicted by AlphaFold (“Confident” and “Very Confident” predictions, pLDDT > 70) were used to visualize probe binding events on all 10 members of the DGK superfamily (Fig. 3 and Fig. S3, ESI†).

For several DGK members, we identified clusters of probe-modified sites that resembled binding regions or pockets for TH211 recognition. For example, we identified a cluster of TH211 binding sites on DGKα located in a binding region that spans the DAGKc and DAGKa regions of the catalytic domain (K384, K543, Y544, K547; Fig. 3(A)). For DGKζ, we observed 2 predicted pockets for probe-recognition that are composed of binding sites in the DAGKc and DAGKa (K311, K473) regions and the DAGKa region (K502, K516, K521, K593; Fig. 3(B)). Docking of TH211 to predicted binding regions showed bound conformations that place the sulfur electrophile in proximity to several nucleophilic residues on DGKs (binding affinities of −7 to −9 kcal mol^−1^ using AutoDock Vina,^44,45^Fig. 3).

Inspection of predicted binding regions on AlphaFold structures of other DGK members identified probe binding profiles that were not concentrated to a specific region but appeared to be more diffuse. For example, the TH211 binding sites detected on DGKι, DGKκ, and DGKθ were located at multiple regions across the predicted structures with no obvious pocket for probe recognition (Fig. S3, ESI†). The lack of a defined binding region was not a general phenomenon of DGK subtypes but appeared to be isoform specific. For example, we identified members of both type 2 (DGKδ *vs.* DGKκ) and 4 DGKs (DGKζ *vs.* DGKι) that contained or lacked a predicted binding region, respectively (Fig. 3 and Fig. S3, ESI†).

### Evaluating C1 domain swaps between type DGK subtypes

We previously developed DGK C1 chimera proteins to exchange C1 domains between type 1 DGKs for evaluating corresponding effects on DGK metabolism in live cells.^12^ These studies identified the C1 domain of type 1 DGKs as important regulators of fatty acid chain specificity. The prominent covalent binding of TH211 to C1 domains of a large fraction of the DGK family presented an opportunity to compare molecular recognition of C1 domains across DGK subtypes through probe binding activity. Specifically, we explored C1 domain swaps across DGK subtypes and the resulting impact on TH211 recognition and biochemical activity of protein engineered chimeras.

**Fig. 4:**
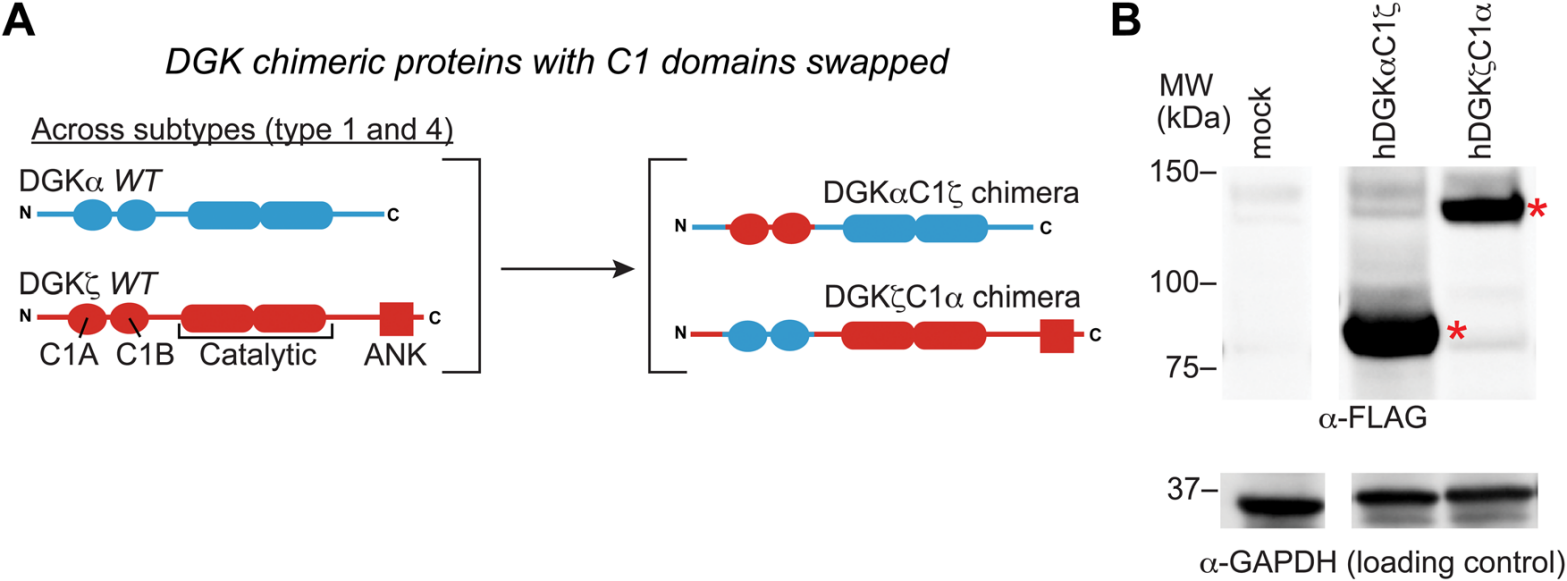
C1 domain exchange between type 1 and 4 DGKs using protein engineering. (A) Schematic of the DGK C1 chimera proteins tested in this study. (B) Expression of DGK chimera proteins was evaluated by western blot using anti-FLAG antibodies. Equivalent protein loading was confirmed by anti-GAPDH. All data shown are representative of *n* = 3 biologically independent experiments. Recombinant proteins are highlighted by red asterisk.

We selected DGKζ for comparative studies with DGKα because expression of these isoforms is enriched in T cells where they function as negative regulators of TCR signaling and are emerging as promising targets for cancer immunotherapy.^13,22,46^ We produced chimeras that evaluated DGKζ C1 domains engrafted into the DGKα protein backbone (DGKαC1ζ) and the corresponding C1 domain swapped counterpart (DGKζC1α, Fig. 4(A)). We confirmed that DGK C1 chimeras were expressed to detectable levels in HEK293T cells using western blots (Fig. 4(B)). See ESI† for protein sequences of DGKαC1ζ and DGKζC1α chimeras.

Next, we treated DGK chimera-expressing HEK293T cells with TH211 (50 μM, 2 h, 37 °C) followed by quantitative chemoproteomics. C1 domain exchange across DGK subtypes 1 and 4 revealed TH211 binding profiles in support of C1 effects on molecular recognition. The lack of TH211 probe modification on Tyr residues in C1 domains of DGKζ was retained when exchanged into the DGKα backbone (DGKαC1ζ, Table S1B, ESI†). Interestingly, covalent binding of TH211 to Lys on DGKζ C1A (K123, K134, K147) and C1B (K189, K194) was largely retained when these domains were engrafted into the DGKα backbone (C1A: K231, K242, K255; C1B: K297, K302; DGKαC1ζ, Table S1B, ESI†). In contrast, TH211 binding to transplanted C1 domains from DGKα appeared to be influenced by the surrounding DGKζ backbone. The specific labeling of C1A (Y240) on wild-type DGKα was replaced by prominent TH211 binding to Tyr and Lys sites of C1A and C1B on DGKζC1α (Table S1B, ESI†).

**Fig. 5:**
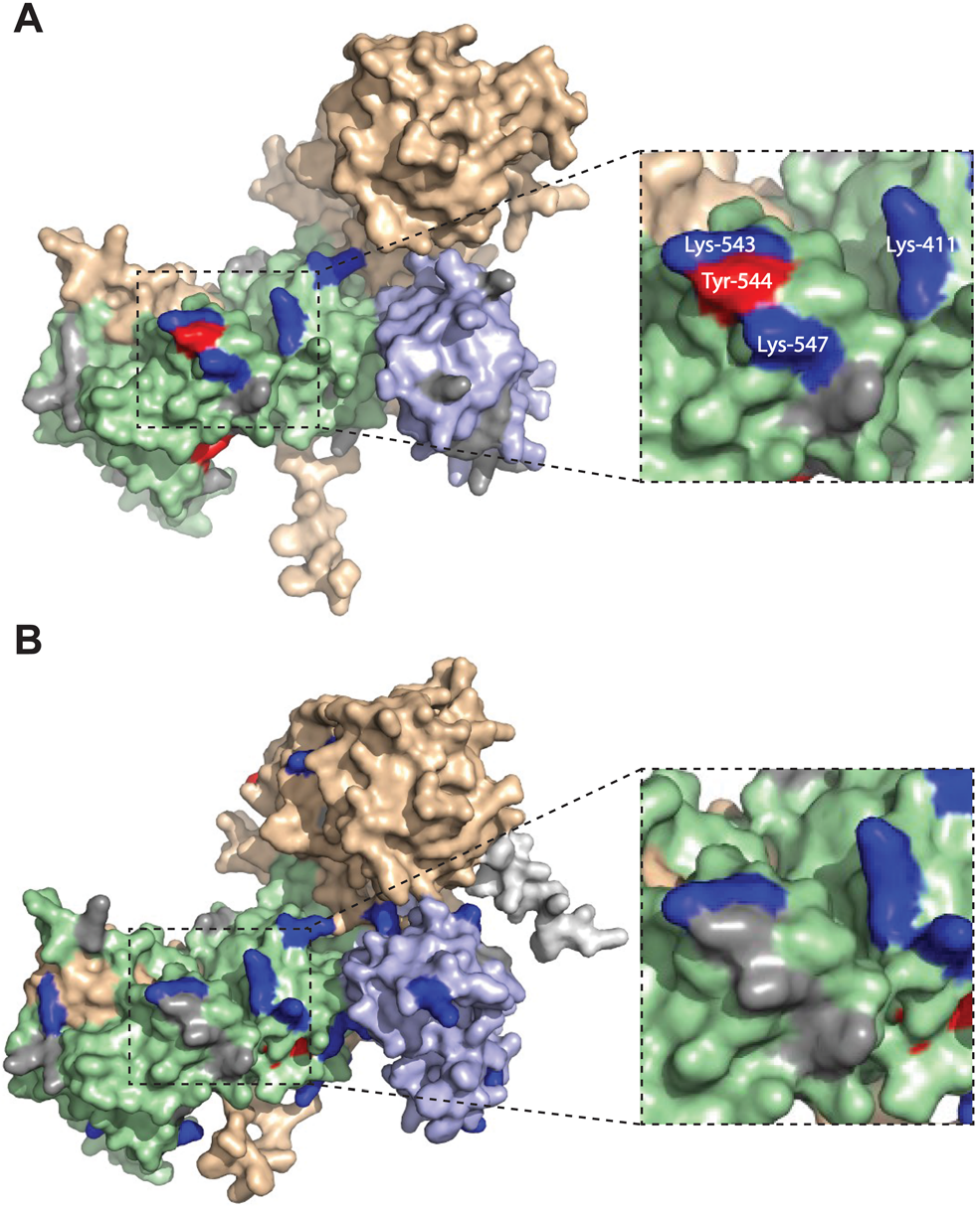
Altered TH211 binding profile in the catalytic domain of C1 domain exchanged DGKα chimera. DGK chimera proteins are composed of the catalytic and regulatory backbone of a DGK protein (DGKα or DGKζ) and tandem C1 domains from a different DGK protein (C1α or C1ζ). TH211 binding to human DGKαC1ζ or DGKζC1α was detected by treatment of recombinant expressing HEK293T cells with TH211 (50 μM) for 2 h at 37 °C followed by quantitative chemoproteomics. (A) DGKα AlphaFold structure with TH211-modified sites highlighted. (B) The predicted chimeric DGKαC1ζ structure generated by ColabFold.^47^ Inset showing expanded region of TH211 bound sites in the catalytic domain of DGKα that are lost with C1 domain exchange. The C1A and C1B domains are shown in light blue. The catalytic domain (DAGKc and DAGKa regions) is shaded in light green. Probe modified Lys and Tyr are shown in dark blue and red, respectively. Lys and Tyr residues confidently predicted by AlphaFold (“Confident” and “Very Confident” predictions, pLDDT > 70) but not modified by our probe are shown in gray. Lys and Tyr residues predicted less confidently (“Low” and “Very Low” predictions, pLDDT < 70) are not highlighted and were not included in the analysis. The FLAG tag is shown in light gray. All data shown are representative of *n* = 3 biologically independent experiments. Predicted structures were visualized using PyMOL.

**Fig. 6:**
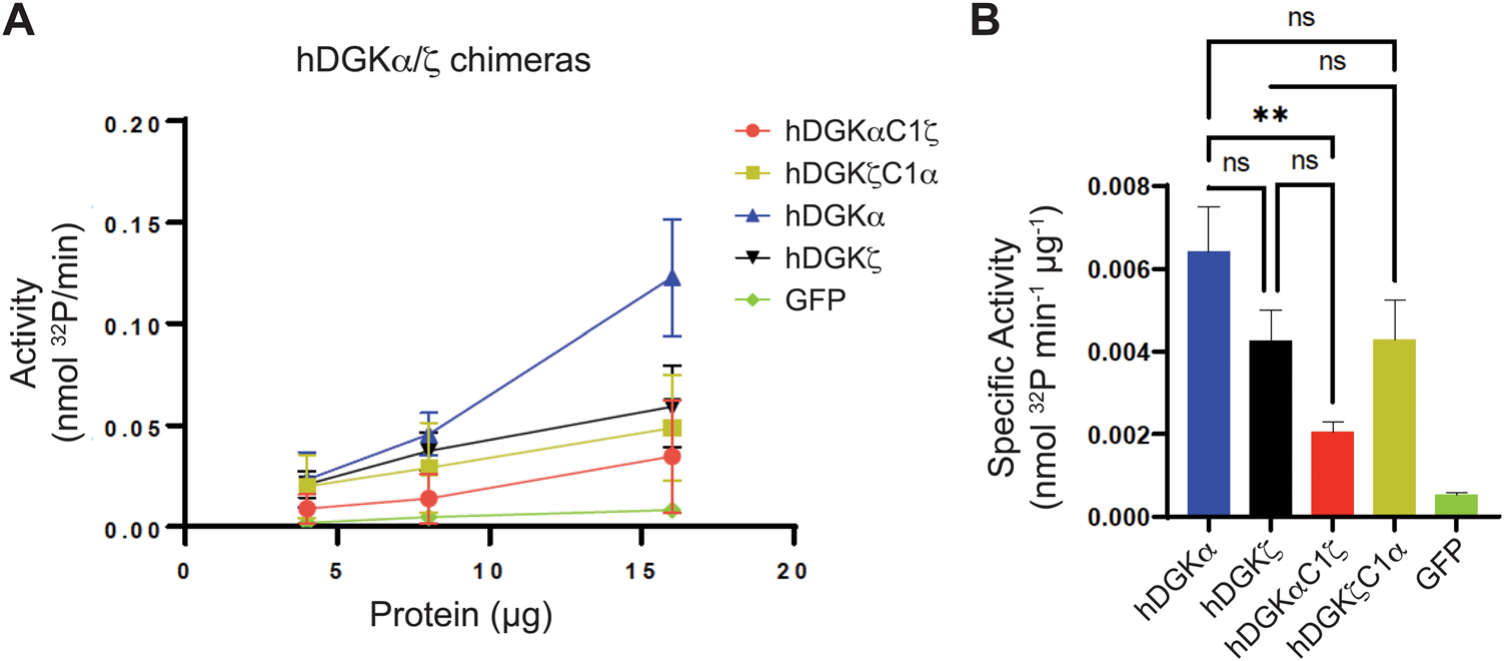
Biochemical evaluation of type 1 and 4 DGK chimeras. (A) The biochemical activity of DGK-α and -ζ wild-type, chimera, and GFP lysate was measured in a dose dependent manner (4, 8, 16 μg of respective lysates) by monitoring ATP incorporation into product as a function of time (nmol ^32^P min^−1^). (B) Average specific activity of each respective lysate calculated from A and normalized to the protein amount used in each assay. **p < 0.01, One-way ANOVA with Tukey's MC correction (*n* = 3 biologically independent experiments).

We mapped the TH211 binding events onto a predicted structure of DGKαC1ζ generated by ColabFold.^47^ We compared the predicted TH211 binding regions on DGKαC1ζ with DGKα and found that, in general, we observed increased overall TH211 binding to the chimera protein but loss of binding in a region that is in proximity to previously reported ATP binding sites (Y544, K547, Fig. 5). These chemoproteomic findings support potential alterations in active site recognition of DGKαC1ζ that we further tested experimentally using biochemical substrate assays. We compared catalytic activity of recombinant DGK chimera-expressing proteomes using a radiolabeled ATP substrate assay in DAG liposomes as previously described.^35,48^ Recombinant overexpression of recombinant wild-type DGKα or DGKζ resulted in a protein concentration dependent increase in DAG phosphorylation activity compared with a GFP-expressing control sample (Fig. 6(A)). Exchange of DGKα C1 domains into the DGKζ backbone construct did not impact catalytic activity of the resulting DGKζC1α chimera protein. In contrast, DGKα was less tolerant of a C1 domain exchange with a subtype 4 DGK. Inserting DGKζ C1 domains into the DGKα backbone produced a chimera protein that displayed significantly reduced catalytic activity compared with wild-type counterpart (0.002 *vs.* 0.006 nmol min^−1^ μg^−1^ for DGKαC1ζ compared with DGKα, respectively; Fig. 6(B)).

Collectively, our findings support crosstalk between regulatory and catalytic domains in molecular recognition of DGK active sites. The loss of covalent binding to TH211 binding pockets in the active site of predicted DGKαC1ζ structures corresponded with loss of biochemical activity for the recombinant chimera protein.

## Discussion

DGKs are metabolic enzymes implicated in regulation of cellular DAG and PA biology.^1–4^ The structural, bioenergetic and signaling roles of these lipids position DGKs as key hubs for supplying general and bespoke metabolites in diverse cellular biology. The existence of ten mammalian DGKs support the potential for functional diversification to tailor lipid metabolism to specific cellular programs. The molecular basis of this substrate specificity remains ill-defined although chemical proteomic studies are providing insights to active site recognition through covalent probe binding.^28^ Here, we deployed sulfonyl-triazole probes containing a DGK-directed binding element (RF001) for establishing covalent binding profiles in cells that can be mapped to AlphaFold structures for predicting small molecule binding pockets across the DGK superfamily.

Our experimental workflow adopted a recombinant DGK overexpression system previously established for lipidomics^12^ and applied here for chemoproteomic evaluation of the DGK superfamily. Importantly, the recombinant protein format ensured detectable levels of all DGK isoforms to facilitate a family-wide evaluation by chemoproteomics. The resulting TH211 binding profiles identified modified sites in the catalytic domain of all DGK members. These results were not surprising given the high primary sequence conservation and the high propensity of this domain to interact with diverse covalent probes.^28^ The restricted probe binding profiles of EF hands and C1 domains within the type 1 DGKs supported these regulatory regions of DGKs as important differentiators of molecular recognition. By expanding TH211 chemical proteomics beyond DGKα to additional isoforms, we were able to identify reactive Tyr and Lys residues in protein–protein interaction motifs including ankyrin repeats (Y841, Y876, K836, K886; DGKζ) and the Ras-association domain of DGKs (Y412, K400, K420; DGKθ, Table S1B, ESI†). The lower frequency of modified Tyr compared with Lys sites suggests that incorporation of the more Tyr chemoselective 1,2,4-sulfonyl triazole electrophile^34^ might be advantageous for enhancing selectivity of DGK covalent inhibitors in future studies.

When overlaid onto AlphaFold structures, the collection of probe-modified sites detected on DGKs could be used to predict binding pockets for a substantial fraction of DGK isoforms. Notably, we identified binding regions that contained clusters of probe-modified sites on DGKα (K384, K543, Y544, K547), DGKζ (K502, K516, K521, K593; K311, K473), DGKγ (K356, Y358, Y535, K542), and DGKδ (K271, K198, K337; Fig. 3 and Fig. S3, ESI†). We find it interesting that the AlphaFold structures of DGKα show the C1 domains juxtaposed to the catalytic domain, which agrees with our previous findings that support an interdomain active site architecture for this DGK isoform^37^ (Fig. 3). We tested this working model further by exchanging C1 domains between DGKα and DGKζ given their key role in T cell activation and the interest in developing isoform-selective inhibitors for cancer immunotherapy.^13,27,35,36,49^ While type 1 DGK C1 domains were moderately interchangeable as previously reported,^12^ our findings here showed the tolerability of C1 domain swaps between subtype 1 and 4 appeared to be isoform specific. The substantial loss of catalytic activity of DGKαC1ζ compared with wild-type DGKα further supports the role of C1 domains for biochemical function of this isoform. Notably, the impaired biochemical activity of DGKαC1ζ correlated with loss of TH211 bound sites in the catalytic domain of DGKα from C1 domain exchange (Fig. 5 and 6).

We recognize that the covalent binding profiles reported here were detected on recombinant but not native DGKs. While important, we note that studies of endogenous proteins will likely require SuTEx probes tailored for individual DGKs to screen and identify appropriate cell and tissue types for chemoproteomic evaluation of native counterparts. We also cannot formally rule out the potential impact of expression differences between recombinant DGKs on the TH211 reactivity profiles observed in cells. Future studies could address this question by comparing TH211 binding to DGK isoforms (recombinant or endogenous) with more comparable expression levels.

We are also cognizant that the RF001 fragment in TH211 is less potent compared with its parent compound ritanserin for DGKα inactivation,^37^ which could indicate the potential for non-specific binding of TH211 to DGKs. While we cannot formally rule out this possibility, we note that the calculated ligand efficiency^50,51^ (LE) for RF001 is enhanced compared with ritanserin (0.24 and 0.18 kcal mol^−1^ per non-H atom for RF001 *vs.* ritanserin, respectively). The higher LE of RF001 combined with inhibitory activity of TH211 across multiple DGK subtypes (Fig. S1, ESI†) support functional profiling of DGKs using TH211. Covalent probe binding, while important as a first step towards establishing function requires complementary biochemical and cell biological assays. Future studies can evaluate the cell biological effects of mutating these residues on lipid metabolism and signaling in recombinant gain of function studies or mutating sites of interest on endogenous DGKs by CRISPR-Cas9.^52^ We envision that the strategy reported here will serve as a guide to prioritize binding sites for mutagenesis, biochemical and eventually cell biological verification of DGK function.

In summary, we present a comprehensive ligand binding map of reactive Tyr and Lys residues that are readily accessible in live cell probe labeling studies and map to predicted small molecule binding pockets across the DGK superfamily. These findings should guide future efforts to explore regulatory and catalytic DGK domains for basic biochemistry and inhibitor discovery efforts.

## Author contributions

Conceptualization, R. M. and K.-L. H.; methodology, R. M. and K.-L. H.; investigation, R. M., M. S., M. C. L., K. Y. and A. H. L.; writing, R. M. and K.-L. H.; funding acquisition, R. M. and K.-L. H.; formal analysis, R. M., M. M. R., D. L. B., M. C. L., T. E. H., data curation, D. L. B., M. M. R. and K.-L. H.; supervision, K.-L. H.

## Conflicts of interest

K.-L. H. is a founder and scientific advisory board member of Umbra Therapeutics.

## Supplementary Material

CB-004-D3CB00057E-s001

CB-004-D3CB00057E-s002
