# Supplementary material for: Predicting small molecule binding pockets on diacylglycerol kinases using chemoproteomics and AlphaFold
Source: RSC Chem Biol. 2023 May 15;4(6):422–30. doi: 10.1039/d3cb00057e (PMC10246554; doi:10.1039/d3cb00057e)
Supplement: CB-004-D3CB00057E-s001 [file CB-004-D3CB00057E-s001.pdf]

Electronic Supplementary Material (ESI) for RSC Chemical Biology.
This journal is © The Royal Society of Chemistry 2023

SUPPORTING INFORMATION

Predicting small molecule binding pockets on diacylglycerol kinases using chemoproteomics and AlphaFold

Roberto Mendez[1], Minhaj Shaikh[1], Michael C. Lemke[2], Kun Yuan[1], Adam H. Libby[1,4], Dina L. Bai[1], Mark M. Ross[1], Thurl E. Harris[2] and Ku-Lung Hsu*[1,2,3,4]

[1]Department of Chemistry, University of Virginia, Charlottesville, Virginia 22904, United States

[2]Department of Pharmacology, University of Virginia School of Medicine, Charlottesville, Virginia 22908, United States

[3]Department of Molecular Physiology and Biological Physics, University of Virginia, Charlottesville, Virginia 22908, United States

[4]University of Virginia Cancer Center, University of Virginia, Charlottesville, VA 22903, United States

*Author to whom correspondence should be addressed:

Present Address:
Email: ken.hsu@austin.utexas.edu (K.-L.H.)
Department of Chemistry
University of Texas at Austin
100 East 24th Street
Austin, TX 78712
Phone: 434-297-4864

CONTENTS:

1. Supporting Figures
2. Methods
3. Appendix
4. Full length blots
5. References

1. SUPPORTING FIGURES

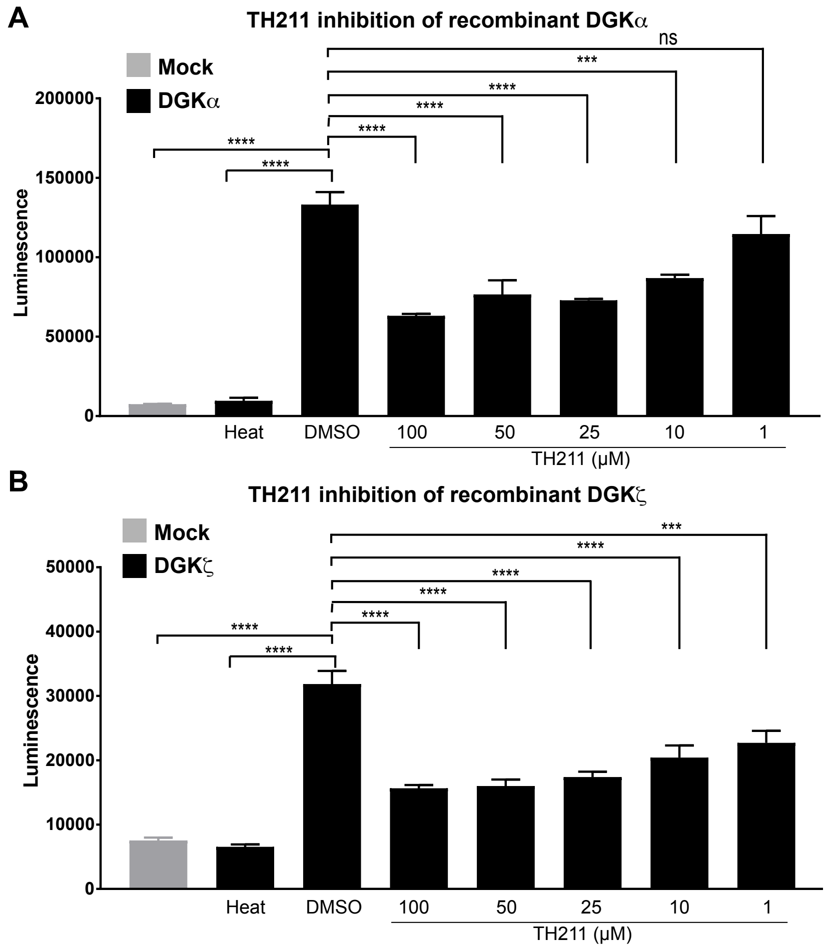

Figure S1. TH211 treatment blocks catalytic activity of recombinant DGKα and DGKζ as determined by ADP-glo substrate assay. Production of active recombinant DGKα- or DGKζ was determined by enhanced activity in DGKα- or DGKζ-HEK293T- versus mock-transfected soluble proteomes as measured using an ADP-glo substrate assay. Specificity of activity to recombinant protein was confirmed by abolishment of the observed activity with heat denatured recombinant lysates (95° C for 5 min). Pretreatment with TH211 (100, 50, 25, 10 and 1 μM) resulted in concentration dependent blockade of recombinant DGKα (A) and DGKζ (B) activity. Data shown are mean +/- SEM for n = 2 independent biological replicates; ****$p \leq 0.0001$, ***$p \leq 0.001$, or not significant (ns) for samples compared with DMSO-treated recombinant DGK lysate group. Statistical significance was determined using a Dunnett multiple comparison following a one-way ANOVA test.

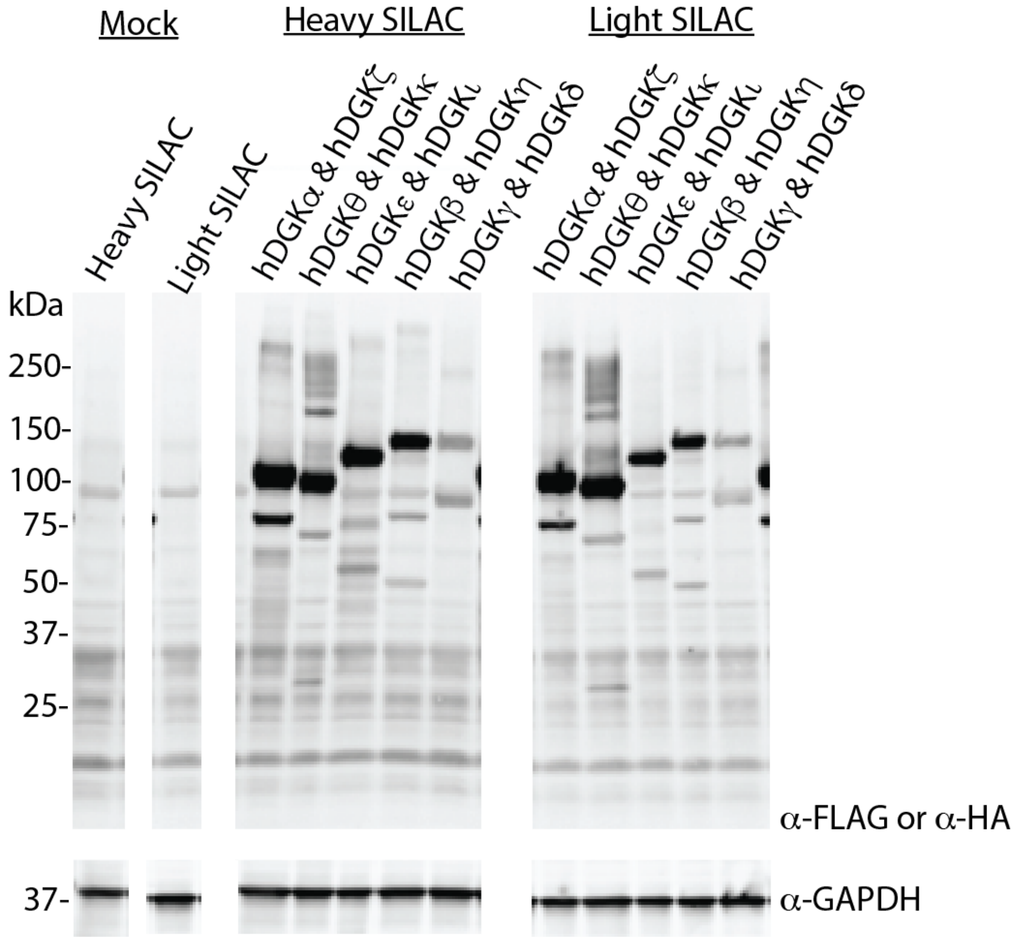

Figure S2. Expression of recombinant DGKs was comparable in SILAC light and heavy HEK293T cells. Recombinant human DGK proteins were co-expressed in SILAC HEK293T cells and used for chemical proteomic evaluation. Expression of recombinant DGKs was detected by western blots using anti-FLAG antibodies except for DGKζ and DGKι, which were detected with anti-HA antibody. Equivalent protein loading was confirmed by anti-GAPDH. Recombinant protein expression was comparable between HEK293T cells cultured in light and heavy media.

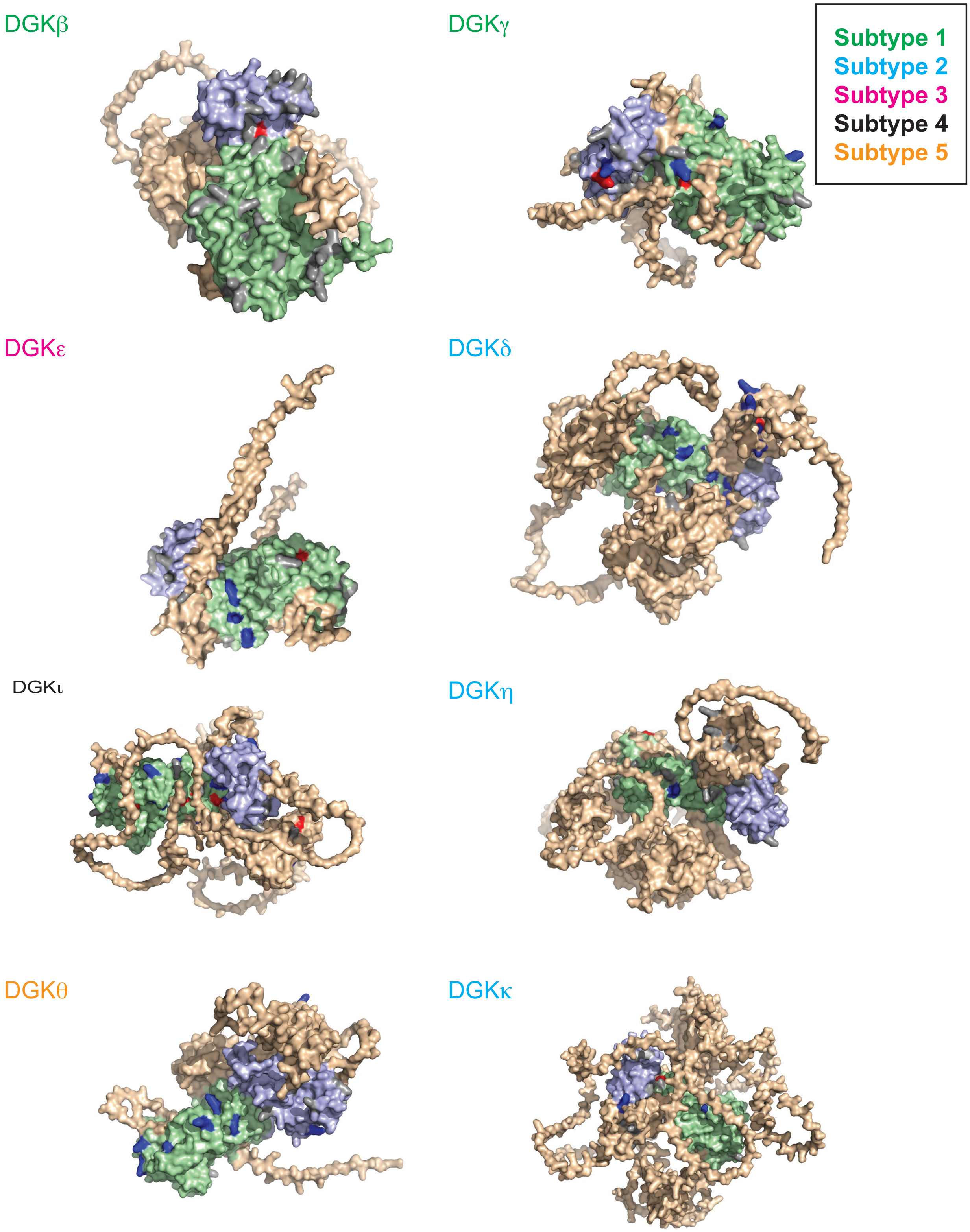
DGKβ
DGKγ
Subtype 1
Subtype 2
Subtype 3
Subtype 4
Subtype 5
DGKε
DGKδ
DGKι
DGKη
DGKθ
DGKκ

Figure S3. Covalent binding to predicted pockets of DGKs in living cells. The TH211 binding sites were detected by live cell treatments of recombinant DGK overexpressed-HEK293T cells with TH211 (50 μM, 2 h, 37 °C) followed by quantitative chemical proteomics. The C1 domains are shaded in light blue. The catalytic domain (DAGKc and DAGKa region) is shaded in light green. Probe modified Lys and Tyr are shown in dark blue and red, respectively. Lys and Tyr residues confidently predicted by AlphaFold ("Confident" and "Very Confident" predictions, pLDDT > 70) but not modified by our probe are shown in gray. Lys and Tyr residues predicted less confidently ("Low" and "Very Low" predictions, pLDDT < 70) are not highlighted and were not included in the analysis. All data shown are representative of 3 experiments (n = 3 biologically independent experiments). Predicted structures were visualized using PyMOL (Version 2.6; https://pymol.org).

2. METHODS

Reagents

TH211 was synthesized and used for live cell chemoproteomics as previously described[1].

Cell culture

HEK293T cells were cultured with corresponding SILAC media supplemented with 10% dialyzed Fetal bovine serum (DFBS, Us. Source, Omega Scientific), 1% L-glutamine (ThermoFisher Scientific), and either ^{12}C, ^{14}N-lysine and arginine (100 μg/mL) or ^{13}C, ^{15}N-lysine and arginine (100 μg/mL) for light and heavy cells, respectively (Sigma-Aldrich). Cells were maintained at 37 °C with 5% CO_2 and used for experiments around 90% confluency.

Transient transfection

Recombinant proteins were produced by transient transfection of HEK293T cells with recombinant DNA as previously described[2]. The following constructs were generated by recombination of Addgene plasmids using the Gateway cloning system (Invitrogen): pGCFlag-DGKγ-FLAG (human) and pGCFlag-DGKκ-FLAG (human). The following plasmids were purchased commercially from GenScript: pcDNA3.1-DGKα-FLAG (human), pcDNA3.1-DGKβ-FLAG (human), pcDNA3.1-DGKε-FLAG (human), pcDNA3.1-DGKη-FLAG (human), pcDNA3.1-DGKθ-FLAG (human), pcDNA3.1-FLAG-DGKζC1α (human) and pcDNA3.1-FLAG-DGKαC1ζ (human). All other vectors were gifted to Dr. Thurl Harris (University of Virginia, School of Medicine) by Dr. Kaoru Goto (Yamagata University, School of Medicine) and

Dr. Fumio Sakane (Chiba University) and were kindly shared with us: pCMV-HA-DGKζ (human, short isoform, Q13574-2), pcDNA3-HA-DGKι (human) and pCMV-7.13xFLAG-DGKδ (human).

Western blot analysis

Western blot analysis of recombinant protein expression was performed as previously described[3].

TH211 covalent binding to DGKs *in situ*

Media was aspirated from recombinant DGK-HEK293T cells when cells reached ~90% confluency (~48 h after transient transfection). Cells were washed gently with PBS and treated with serum-free media containing DMSO vehicle or TH211 probe (50 μM final; 50X stock in DMSO). Cells were returned to the incubator for 2 h at 37 °C. After removal of media, cells were washed with cold PBS 2X and then harvested into 15 mL conical tubes. Samples were pelleted by centrifugation (500 x *g*, 5 min, 4 °C) to remove PBS. Samples were resuspended in 500 μL of PBS containing protease and phosphatase inhibitor (EDTA-free) and transferred to 1.5 mL Eppendorf tubes. Samples were sonicated (1 sec, 20% amp, 3x). Protein concentrations were determined by Bio-Rad DC protein assay and sample concentrations adjusted to 2 mg/mL. Samples were snap frozen in liquid N_2 and stored at -80 °C until analyzed.

SILAC sample preparation for MS-based chemical proteomic assay

Light and heavy proteomes from TH211-treated cells were prepared for LC-MS chemical proteomic assay as previously described[1].

LC-MS/MS chemical proteomics

The enriched probe-modified peptide samples were analyzed by LC-MS/MS using an Easy-nLC 1200 (Thermo Scientific) coupled with an Orbitrap Q Exactive Plus mass spectrometer (Thermo Scientific) as previously described[1]. Identification of peptides and target proteins from the LC-MS/MS raw data was accomplished as previously described[1]. Data were searched using Byonic and Skyline. Byonic search parameters were as follows: up to 3 missed cleavages, 10 ppm precursor mass tolerance, 50 ppm fragment mass tolerance, too high (narrow) 'precursor isotope off by x', precursor and charge assignment computed from MS1, maximum of one precursor per MS2 and 1% protein false discovery rate. Variable common modifications included methionine oxidation (+15.9949 Da), SILAC labels on arginine and lysine (+10.0083 Da and +8.0142 Da respectively), and our sulfonyl probe modification on tyrosine and lysine (+635.2737). A fixed common modification of carbamidomethylation on cysteine (+57.0215 Da) was also included. Isoform data were searched using a modified human protein database (UniProt human database 06/2019, angiotensin I and vasoactive intestinal peptides standards). For the chimeric data sets, the plasmid DNA sequences were converted to proteins sequences using Benchling (Benchling Biology Software 2022, retrieved from https://benchling.com) and added to the modified database. Results from Byonic and Skyline were combined and filtered in R to retain high confidence peptides as determined by the following criteria: Byonic score ≥ 500, a precursor mass error within 5 ppm; normalized SILAC ratio (SR) > 5, with both isotope dot-product (iDOTP) and ratio dot-product (rDOTP) ≥ 0.8. These results were used for all analyses except the generation of Table S1, which was based on Byonic analysis only and the results were filtered using these criteria: Score ≥ 300, precursor mass error within 5 ppm, Delta ≥ 25, DeltaMod Score ≥ 20 and Log Prob ≥ 3.0.

Biochemical substrate assay of DGK chimeras

A liposomal substrate assay for measuring DAG kinase activity was performed as previously described[4-6]. In brief, each reaction, performed in triplicate, comprised of buffer B, 1 mM DTT, 0.1 mM $CaCl_2$, 2 mM lipids as well as each respective lysate overexpressing either DGK or GFP as control. Due to relative expression differences between separate preparations, the amount of lysate protein used in each reaction was confirmed to fall within the linear range of detection (4, 8 and 16 μg for each DGK and GFP control) to account for the variability in enzyme activity due to transfection efficiency. Each reaction received 10 μL of 10 mM ATP spiked with [γ^{32}P]-ATP (100 μL final volume) and incubated at 30 °C for 20 min. 0.5 mL of methanol with 0.1 N HCl was used to quench each reaction, followed by 1 mL of ethyl acetate and 1 mL of 1 M $MgCl_2$ for organic phase separation. Each reaction was vortexed, and 0.5 mL of the organic phase was removed. The incorporation of [^{32}P] into DAG was measured from these organic phase samples via scintillation counter. Each construct's specific activity was calculated as average nmol of product per minute per μg of total lysate protein.

ADP-glo substrate assay of TH211 inhibition

TH211 inhibitory activity against recombinant DGKα- or DGKζ-HEK293T soluble proteomes was evaluated by ADP-glo following published methods[2] except TH211 pretreatment with lysate was performed in the presence of free ATP (55 μM) in reaction buffer.

3. APPENDIX

Human DGKα and DGKζ chimera construct plasmids were custom synthesized by GenScript. The sequences are shown below - DGKα (isoform 1) sequence, DGKζ (long isoform, Q13574-1) sequence, FLAG-tag insert:

Human_DGKαC1ζ_FLAG_pcDNA3.1(+)

MDYKDDDDKEFTMAKERGLISPSDFAQLQKYMEYSTKKVSDVLKLFEDGEMAKYVQGDAIGY
EGFQQFLKIYLEVDNVPRHLSLALFQSFETGHCLNETNVTKDVVCLNDVSCYFSLLEGGRPEDKL
EFTFKLYDTDRNGILDSSEVDKIILQMMRVAEYLDWDVSELRPILQEMMKEIDYDGSGSVSQAE
WVRAGATTVPLLVLLGLEMTLKDDGEHIWFETNVSGDFCYVGEQYCVARMLKSVSRRKCAAC
KIVVHTPCIEQLEKINFRCKPSFRESGSRNVREPTFVRHHWVHRRRQDGKCRHCGKGFQQKFTFH
SKEIVAISCSWCKQAYHSKVSCFMLQQIEEPCSLGLRDHILPPSSIYPSVLASGPDRKNSKTSQKT
MDDLNLSTSEALRIDPVPNTHPLLVFVNPKSGGKQGQRVLWKFQYILNPRQVFNLLKDGPEIGLR
LFKDVPDSRILVCGGDGTVGWILETIDKANLPVLPPVAVLPLGTGNDLARCLRWGGGYEGQNLA
KILKDLEMSKVVHMDRWSVEVIPQQTEEKSDPVPFQIINNYFSIGVDASIAHRFHIMREKYPEKFN
SRMKNKLWYFEFATSESIFSTCKKLEESLTVEICGKPLDLSNLSLEGIAVLNIPSMHGGSNLWGDT
RRPHGDIYGINQALGATAKVITDPDILKTCVPDLSDKRLEVVGLEGAIEMGQIYTKLKNAGRRLA
KCSEITFHTTKTLPMQIDGEPWMQTPCTIKITHKNQMPMLMGPPPRSTNFFGFLS

Human_DGKζC1α_FLAG_pcDNA3.1(+)

MDYKDDDDKEFTMETFFRRHFRGKVPGPGEGQQRPSSVGLPTGKARRRSPAGQASSSLAQRRRS
SAQLQGCLLSCGVRAQGSSRRRSSTVPPSCNPRFIVDKVLTPQPTTVGAQLLGAPLLLTGLVGMN
EEEGVQEDVVAEASSAIQPGTKTPGPPPPRGAQPLLPLPRYLRRASSHLLPADAVYDHALWGLH
GYYRRLSQRRPSGQHPGPGGRRASGTTAGTMLPTRVRPLSRRRQVALRRKAAGPQAWSALLAK
AITKSGLQHLAPPPPTPGAPCSESERQIRSTVDWSESATYGQHMWRPKRFPRPVYCNLCESSIGLG
KQGLSCNLCKYTVHDQCAMKALPCEVSTYAKSRKDIGVQSHVWVRGGCESGRCDRCQKKIRIY
HSLTGLHCVWCHLEIHDDCLQAVGHECDCGLHAAVVIPPTWILRARRPQNTLKASKKKKRASFK
RKSSKKGPEEGRWRPFIIRPTPSPLMKPLLVFVNPKSGGNQGAKIIQSFLWYLNPRQVFDLSQGGP
KEALEMYRKVHNLRILACGGDGTVGWILSTLDQLRLKPPPPVAILPLGTGNDLARTLNWGGGYT
DEPVSKILSHVEEGNVVQLDRWDLHAEPNPEAGPEDRDEGATDRLPLDVFNNYFSLGFDAHVTL
EFHESREANPEKFNSRFRNKMFYAGTAFSDFLMGSSKDLAKHIRVVCDGMDLTPKIQDLKPQCV
VFLNIPRYCAGTMPWGHPGEHHDFEPQRHDDGYLEVIGFTMTSLAALQVGGHGERLTQCREVV
LTTSKAIPVQVDGEPCKLAASRIRIALRNQATMVQKAKRRSAAPLHSDQQPVPEQLRIQVSRVSM
HDYEALHYDKEQLKEASVPLGTVVVPGDSDLELCRAHIERLQQEPDGAGAKSPTCQKLSPKWCF
LDATTASRFYRIDRAQEHLNYVTEIAQDEIYILDPELLGASARPDLPTPTSPLPTSPCSPTPRSLQGD
AAPPQGEELIEAAKRNDFCKLQELHRAGGDLMHRDEQSRTLLHHAVSTGSKDVVRYLLDHAPP
EILDAVEENGETCLHQAAALGQRTICHYIVEAGASLMKTDQQGDTPRQRAEKAQDTELAAYLEN
RQHYQMIQREDQETAV

4. FULL LENGTH BLOTS

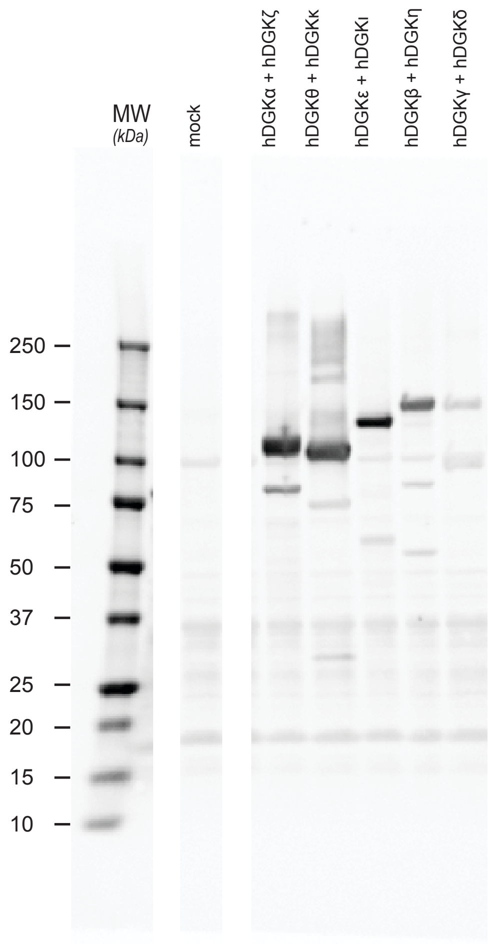

Full images of blots for Figure 1. **Anti-FLAG and -HA shown**.

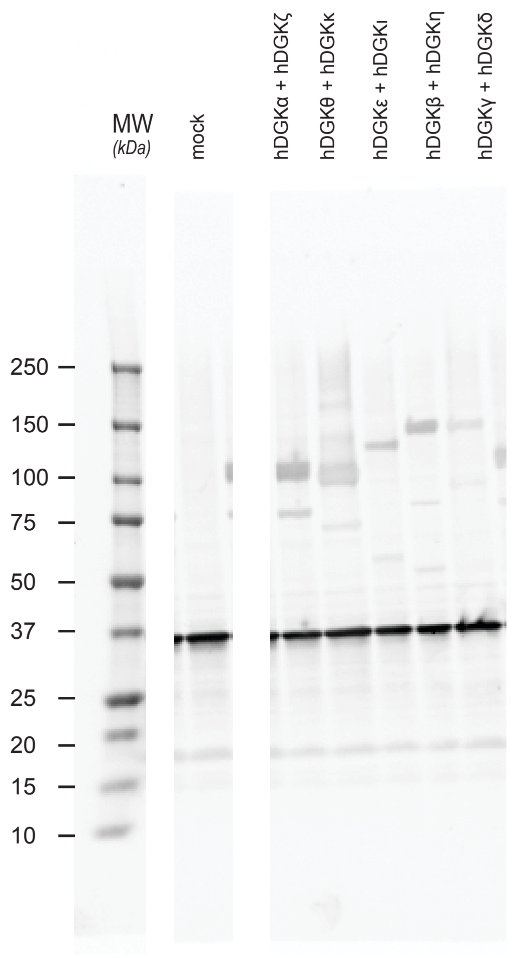

Full images of blots for Figure 1. **Anti-GAPDH shown**.

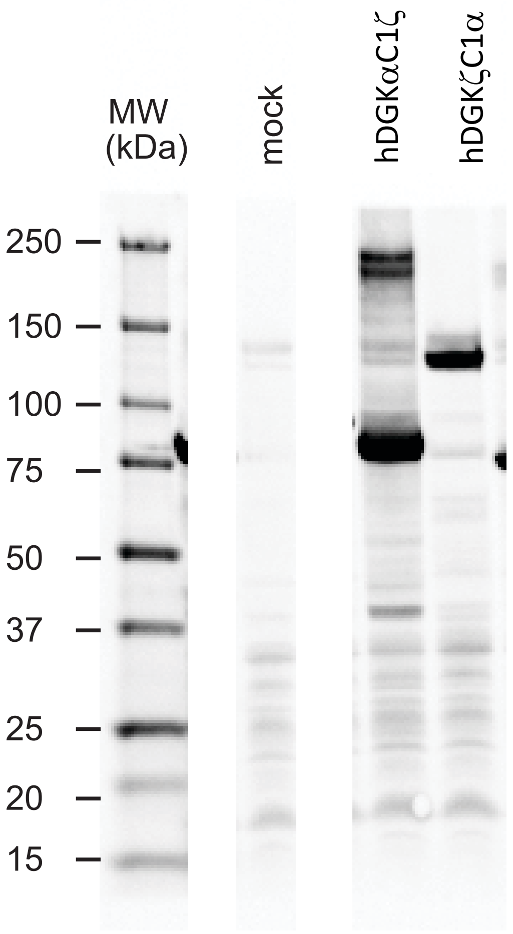

Full images of blots for Figure 4. **Anti-FLAG shown**.

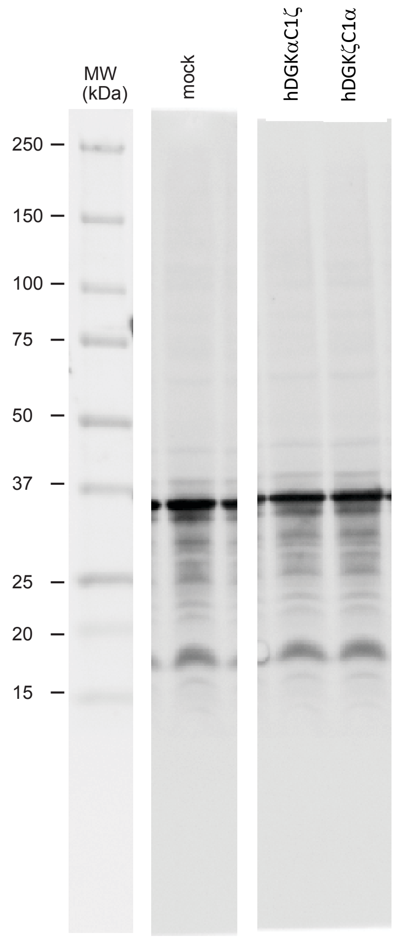

Full images of blots for Figure 4. **Anti-GAPDH shown**.

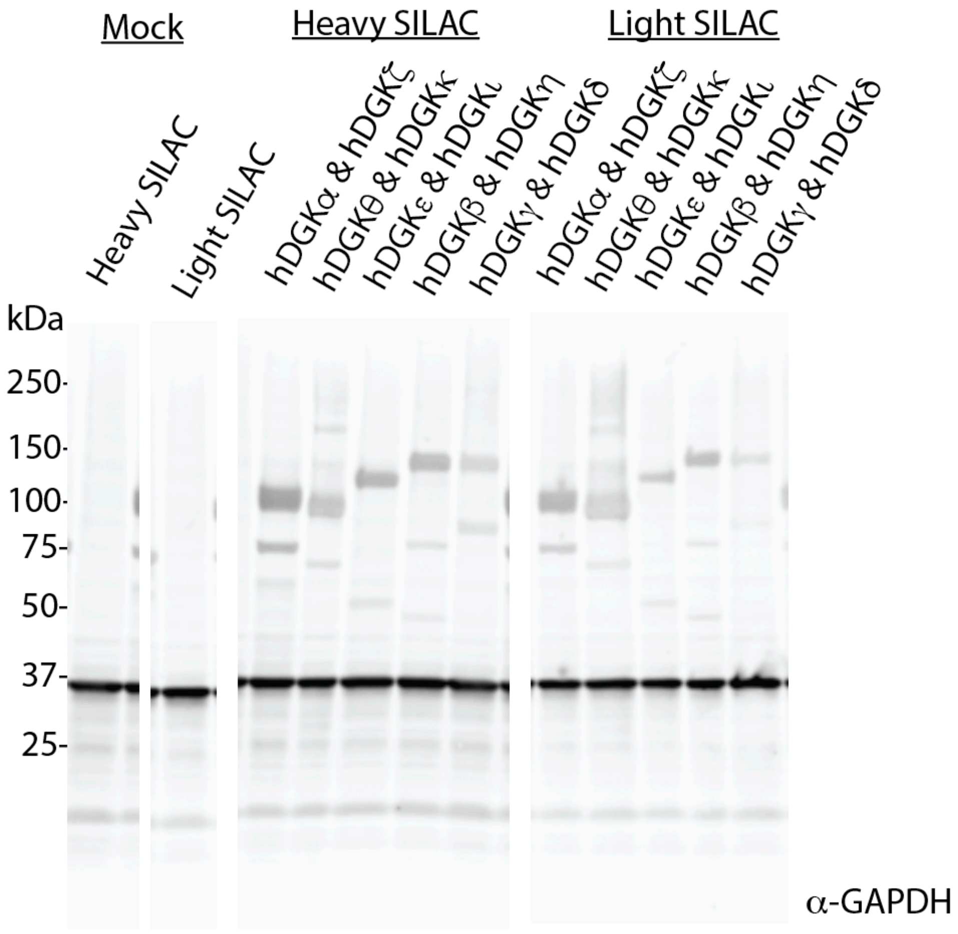

Full images of blots for Figure S2. Anti-GAPDH shown.

5. REFERENCES

1. T. Huang, S. Hosseinibarkooie, A. L. Borne, M. E. Granade, J. W. Brulet, T. E. Harris, H. A. Ferris and K.-L. Hsu, *Chem Sci*, 2021, **12**, 3295-3307.
2. C. E. Franks, S. T. Campbell, B. W. Purow, T. E. Harris and K. L. Hsu, *Cell Chem Biol*, 2017, **24**, 870-880 e875.
3. E. K. Toroitich, A. M. Ciancone, H. S. Hahm, S. M. Brodowski, A. H. Libby and K. L. Hsu, *Chembiochem*, 2021, **22**, 2134-2139.
4. S. Boroda, M. Niccum, V. Raje, B. W. Purow and T. E. Harris, *Biochem Pharmacol*, 2017, **123**, 29-39.
5. M. E. Granade and T. E. Harris, *Methods Enzymol*, 2018, **607**, 373-388.
6. T. B. Ware, C. E. Franks, M. E. Granade, M. Zhang, K. B. Kim, K. S. Park, A. Gahlmann, T. E. Harris and K. L. Hsu, *Nat Chem Biol*, 2020, **16**, 170-178.
